# Synergistic Effects of Combination Therapy with AKT and mTOR Inhibitors on Bladder Cancer Cells

**DOI:** 10.3390/ijms21082825

**Published:** 2020-04-18

**Authors:** Hyera Kim, Su Jin Lee, In Kyoung Lee, Suejean C. Min, Hyun Hwan Sung, Byong Chang Jeong, Jeeyun Lee, Se Hoon Park

**Affiliations:** 1 Division of Hematology-Oncology, Department of Medicine, Samsung Medical Center, Sungkyunkwan University School of Medicine, Seoul 06351, Korea; hyerakim9465@gmail.com (H.K.);; 2 Division of Hematology-Oncology, Department of Internal Medicine, Keimyung University Dongsan Hospital, Daegu 42601, Korea; 3 Division of Hematology-Oncology, Department of Internal Medicine, Ewha Womans University College of Medicine, Seoul 07804, Korea; 4 Department of Urology, Samsung Medical Center, Sungkyunkwan University School of Medicine, Seoul 06351, Korea

**Keywords:** bladder cancer, AKT, mTOR, AZD5363, AZD2014, BEZ235

## Abstract

Despite comprehensive genomic analyses, no targeted therapies are approved for bladder cancer. Here, we investigate whether a single and combination therapy with targeted agents exert antitumor effects on bladder cancer cells through genomic alterations using a three-dimensional (3D) high-throughput screening (HTS) platform. Seven human bladder cancer cell lines were used to screen 24 targeted agents. The effects of 24 targeted agents were dramatically different according to the genomic alterations of bladder cancer cells. BEZ235 (dual phosphatidylinositol-3-kinase (PI3K)/mammalian target of rapamycin (mTOR) inhibitor) showed antitumor effects against most cell lines, while AZD2014 (mTOR inhibitor) had an IC50 value lower than 2 μM in 5637, J82, and RT4 cell lines. AZD5363 (protein kinase B (AKT) inhibitor) exerted antitumor effects on 5637, J82, and 253J-BV cells. J82 cells (PI3KCA and mTOR mutations) were sensitive to AZD5363, AZD2014, and BEZ235 alone or in AZD5363/AZD2014 and AZD5363/BEZ235 combinations. Although all single drugs suppressed cell proliferation, the combination of drugs exhibited synergistic effects on cell viability and colony formation. The synergistic effects of the combination therapy on the PI3K/Akt/mTOR pathway, apoptosis, and EMT were evident in Western blotting. Thus, the 3D culture-based HTS platform could serve as a useful preclinical tool to evaluate various drug combinations.

## 1. Introduction

Bladder cancer is the most common malignancy of the urinary tract with 430,000 newly diagnosed cases in 2012. It is the 13th most common cause of cancer-related death worldwide [1]. Although patients with muscle-invasive bladder cancer (MIBC) are treated with radical cystectomy and platinum-based combination chemotherapy, the 5-year survival rate is only 60% [2,3]. In addition, patients with unresectable or metastatic bladder cancer who received standard chemotherapy had a poor overall survival (OS) of 15 months, owing to the limited treatment options [4,5]. Immune checkpoint inhibitors have shown better safety profiles and have improved survival outcomes as compared to chemotherapy in limited patients overexpressing programmed death-ligand 1 [6,7]. Therefore, the development of a novel targeted therapy for patients with bladder cancer is urgently needed.

Recent advances in genome sequencing techniques have improved our understanding about bladder cancer and provided a more comprehensive approach to study diverse molecular features of bladder cancer [8]. The phosphoinositide 3 kinase (PI3K)/protein kinase B (AKT)/mammalian target of rapamycin (mTOR) pathway is the key driver of carcinogenesis and progression in bladder cancer [9]. The PI3K signaling cascade continues through the activation of AKT, leading to the stimulation of mTOR. In The Cancer Genome Atlas (TCGA) data, 42% of 131 high-grade MIBC patients had mutations, copy number alterations, or RNA expression changes, all of which affected this pathway [10], and 25% of 412 MIBC patients showed PI3K alterations [8].

The PI3K/AKT/mTOR pathway is a potential therapeutic target for bladder cancer. Ongoing trials are designed using a combination of pathway inhibitors or dual target inhibitors to increase the treatment response in bladder cancer [11,12,13]. Although our knowledge has improved on the underlying mechanism, no targeted therapies have been approved for bladder cancer in the last three decades. Therefore, it is imperative to identify the most effective combination of targeted agents and appropriate candidates among patients with diverse genetic profiles in preclinical research.

In the present study, we investigated whether different targeted agents exert antitumor effects against bladder cancer cells, through genomic alterations, using a three-dimensional (3D) high-throughput screening (HTS) platform. In particular, we focused on the effect of AZD5363 (an AKT inhibitor), AZD2014 (an mTOR inhibitor), and BEZ235 (a dual PI3K/mTOR inhibitor) alone or in combination on two bladder cancer cell lines differing in their mTOR mutation status. We also assessed the expression patterns of the markers of the PI3K/AKT/mTOR pathway (p-AKT, p-mTOR, p-S6), apoptosis (poly (ADP-ribose) polymerase (PARP), cleaved caspase-3, B cell lymphoma-2 (Bcl-2), Bax), and epithelial-to-mesenchymal transition (EMT) (phosphor-signal transducer and activator of transcription 3 (p-STAT3), E-cadherin, snail, vimentin) by Western blotting.

## 2. Results

### 2.1. Three Dimensional HTS Platform for Bladder Cancer Cell Lines and Drug Screening

The molecular characteristics of seven bladder cancer cell lines were determined through cBioPortal (www.cbioportal.org) and the Cancer Cell Line Encyclopedia (portals.broadinstitute.org/ccle) and are presented in Table 1. Seven bladder cancer cell lines showed variability in terms of molecular alterations. J82 cells had various mutations of *TP53*, *FGFR3*, *mTOR*, *PI3KCA*, *RB1*, and *RET* genes, whereas 253J-BV cells carried *PI3KCA* and *ERBB4* mutations.

**Figure 1:**
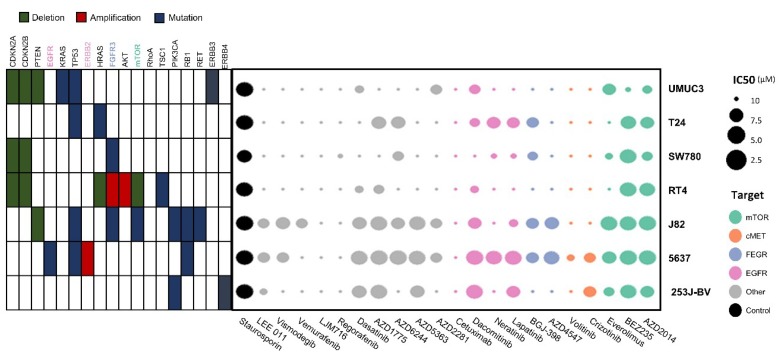
Molecular alterations in cell lines and IC50 values for each drug illustrated as a bubble chart. Using six doses per drug in six replicates, the dose response curves and corresponding IC50 values (μM) were calculated from the scanned images using the S+ Chip Analyzer.

In the process of 3D HTS for drug screening, all seven cell lines were successfully cultured and incubated. Double micropillar chips were exposed to 24 drugs in seven bladder cancer cell lines. Using six doses per drug in six replicates, dose response curves and corresponding IC50 values were calculated from the scanned images using the S+ Chip Analyzer (Samsung Electro-Mechanics Company, Ltd., South Korea). Both molecular alterations in each cell line and IC50 levels of each drug are illustrated as a bubble chart (Figure 1).

The effects of 24 targeted agents were dramatically different according to the genomic alterations of bladder cancer cell lines. BEZ235 (dual PI3K/mTOR inhibitor) exerted antitumor effects against most cell lines except UMUC3 cells. Another mTOR inhibitor, AZD2014 (inhibitor of mTORC1 and mTORC2), had an IC50 value lower than 2 μM in three cell lines (5637, J82, and RT4). The AKT inhibitor AZD5363 exhibited antitumor effects against three cell lines (5637, J82, and 253J-BV).

### 2.2. Effects of the PI3K/AKT/mTOR Targeted Therapy on Bladder Cancer Cells

**Figure 2:**
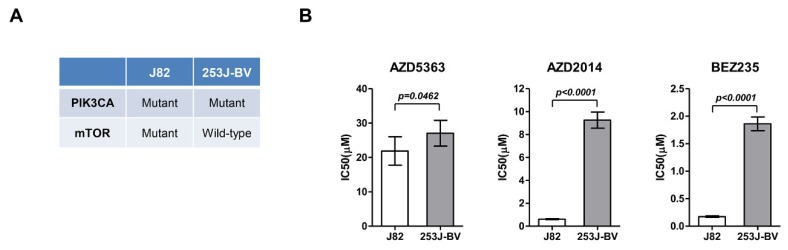
Effects of an AKT inhibitor (AZD5363) and mTOR inhibitors (AZD2014 and BEZ235) on the proliferation of mTOR-mutated or wild-type bladder cancer cells. (**A**) Molecular characteristics of J82 and 253J-BV cell lines. (**B**) Effects of AZD5363, AZD2014, and BEZ235 on J82 and 253J-BV cells were determined using CellTiter Glo. The results are presented as the mean ± SD of triplicate wells and are representative of three independent experiments.

Based on the drug screening results, J82 and 253J-BV cells were cultured, and their viability was evaluated after treatment with AZD5363, AZD2014, and BEZ235. In J82 cells, the IC50 value was 21.865 ± 4.132, 0.617 ± 0.044, and 0.175 ± 0.013 μM for AZD5363, AZD2014, and BEZ235, respectively. The IC50 value of AZD5363, AZD2014, and BEZ235 was 27.038 ± 3.733, 9.254 ± 0.703, and 1.860 ± 0.125 μM, respectively, in 253J-BV cells. J82 cells had a significantly lower IC50 level than 253J-BV cells (Figure 2).

**Figure 3:**
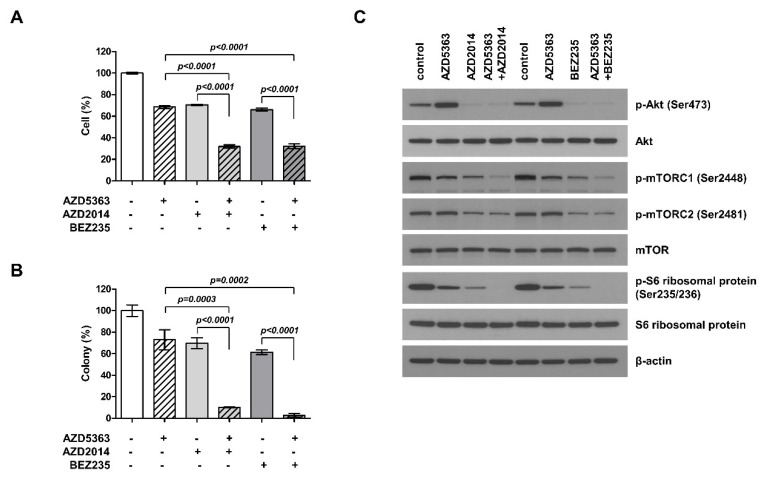
Effects of the combination of an AKT inhibitor (AZD5363) and mTOR inhibitors (AZD2014 and BEZ235) on the proliferation of PI3KCA and mTOR-mutated bladder cancer cells. (**A**) The combination therapy with AZD5363 (3 μM) and mTOR inhibitors (1 μM AZD2014 and 0.5 μM BEZ235) exerted synergistic inhibitory effects on the growth of J82 cells. (**B**) The effects of the combination therapy on J82 cells were determined using the colony formation assay. (**C**) The effects of the combination therapy on PI3K/AKT/mTOR signaling pathway in J82 cells were detected by Western blot analysis. Cells were treated with drugs for 24 h.

To understand the potential effect of the combination therapy targeting the PI3K/AKT/mTOR pathway in PI3KCA- and mTOR-mutated cells, J82 cells were treated with AZD5363, AZD2014, and BEZ235 alone or as AZD5363/AZD2014 and AZD5363/BEZ235 combinations. Although the treatment with all of the three drugs alone could suppress cell proliferation, the combination of drugs elicited higher inhibitory effects on cell viability and colony formation (Figure 3A,B). These results demonstrate that the combination of targeted therapy for this pathway had a synergistic effect against bladder cancer cells carrying both PI3KCA and mTOR mutations. We studied the inhibitory effect on each step within the PI3K/AKT/mTOR pathway. The cell lines treated with the combination therapy showed a decrease in phosphorylation of ATK, mTOR, and S6 (Figure 3C). Thus, these drugs could exert antitumor effects through inhibition of PI3K/AKT/mTOR signaling.

To compare the efficacy of these drugs in bladder cancer cells differing in their mutation status, we studied 253J-BV (PI3KCA-mutated and mTOR wild-type) cells under the same setting. We found that the combination therapy exerted an additive inhibitory effect on cell viability and colony formation, but the efficacy was lower than that seen in J82 cells with PI3KCA and mTOR mutations (Supplemental Materials, Figures S1 and S2).

### 2.3. Effects of PI3K/AKT/mTOR Inhibition on the Apoptosis and EMT of Bladder Cancer Cells

**Figure 4:**
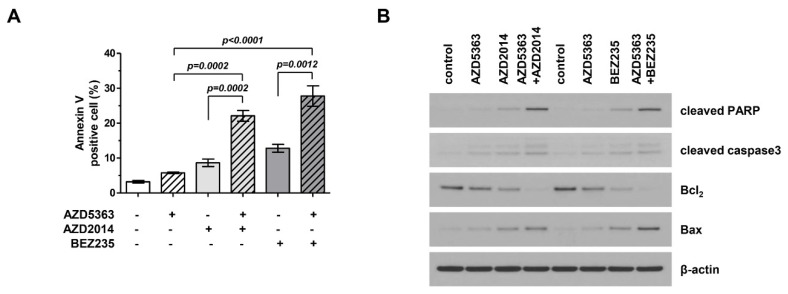
Effects of the combination therapy with an AKT inhibitor (AZD5363) and mTOR inhibitors (AZD2014 and BEZ235) on the apoptosis of PI3KCA and mTOR-mutated bladder cancer cells. (**A**) The combination therapy increased the apoptosis of J82 cells. Apoptosis was detected using Annexin V/propidium iodide staining. Cells were exposed to AZD5363 (3 μM), mTOR inhibitors (1 μM AZD2014 and 0.5 μM BEZ235), or a combination of both for 48 h. (**B**) Pro-apoptotic protein expression was detected by Western blotting. J82 cells were treated with AZD5363 and mTOR inhibitors for 48 h. Proteins were extracted and Western blotting was performed for cleaved PARP, cleaved caspase-3, Bcl-2, and Bax. β-Actin was used as the loading control.

We investigated the apoptotic effects of AZD5363, AZD2014, and BEZ235 on J82 cells. Apoptotic cells were stained using Annexin V and propidium iodide and analyzed by fluorescence-activated cell sorting (FACS). Figure 4A shows that drugs targeting the PI3K/AKT/mTOR pathway promoted cellular apoptosis and that the combination therapy was more effective in inducing apoptosis than treatment with a single agent. We performed Western blot analysis and compared the expression of important signaling proteins involved in apoptosis among cells exposed to different drugs. Figure 4B shows that the expression level of cleaved PARP and Bax increased, that of Bcl-2 level decreased, and the activity of caspase-3 increased. This trend was more prominent in cells treated with the combination therapy.

**Figure 5:**
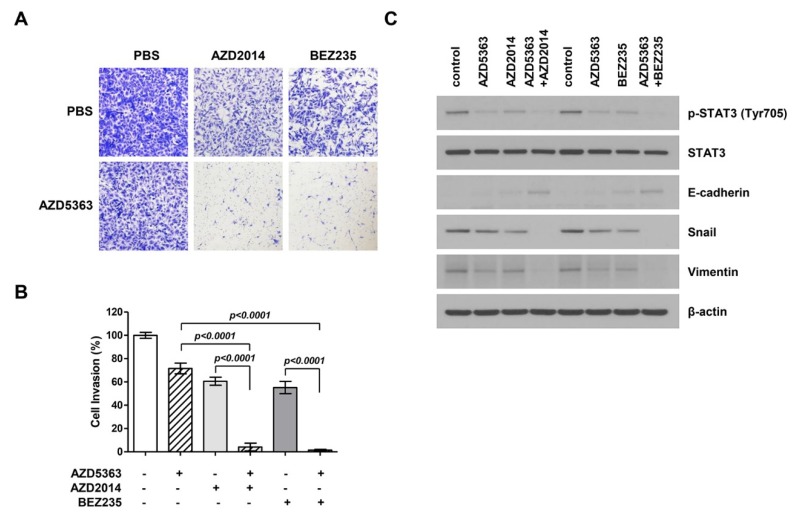
Effects of the combination therapy with an AKT inhibitor (AZD5363) and mTOR inhibitors (AZD2014 and BEZ235) on the invasion and expression of epithelial-to-mesenchymal transition (EMT)-regulating genes of PI3KCA and mTOR-mutated bladder cancer cells. (**A**) Representative images of cell invasion. J82 cells were treated with AZD5363 (3 μM), an mTOR inhibitor (1 μM AZD2014 or 0.5 μM BEZ235), or the combination of both for 48 h. (**B**) Quantification of invasive cells. Combination therapy decreased cell invasion and EMT. Cells that invaded through the membrane were stained with crystal violet and directly counted under a microscope. Data represent mean ± SD of three independent experiments. (**C**) The expression of EMT-related markers was detected by Western blotting. J82 cells were treated with AZD5363 and mTOR inhibitors (AZD2014 and BEZ235) for 48 h. Proteins were extracted and Western blotting was performed using p-STAT3, E-cadherin, snail, and vimentin antibodies. β-Actin was used as the loading control.

To investigate the effect of AZD5363, AZD2014, and BEZ235 on the EMT process in bladder cancer cells, a modified Boyden chamber cell invasion assay was performed using J82 cells. Figure 5A shows that the invasion ability of cancer cells treated with drugs decreased and that the combination therapy exerted stronger effects on cancer cell invasion. A significant difference was observed in the invasion ability of cells treated with single and combination therapy (Figure 5B). To verify the anti-invasion effect of drugs, we performed Western blot analysis to investigate the inhibition of EMT, an important event in cell invasion and migration. The expression levels of migration-related proteins, including p-STAT3, E-cadherin, snail, and vimentin, were assessed. As a result, we found that the expression of p-STAT3 in the EMT-related signaling pathway and mesenchymal markers such as snail and vimentin decreased and that of epithelial markers such as E-cadherin increased (Figure 5C). These effects were more evident in the cells treated with the combination therapy.

## 3. Discussion

In the present study, we evaluated the effects of 24 targeted agents against seven bladder cancer cell lines within 7 days using a 3D HTS platform. As a result, the effects were dramatically different according to the genomic alterations of bladder cancer cells. We demonstrated that combination therapy with an AKT inhibitor (AZD5363) and an mTOR inhibitor (AZD2014 or BEZ235) exerted a synergistic effect on the viability and colony formation ability of bladder cancer cells carrying PI3KCA and mTOR mutations. Further, the effect of AKT and mTOR inhibitors on the PI3K/Akt/mTOR pathway, apoptosis, and EMT was evident in Western blotting.

AZD5363 (capivasertib), a powerful selective inhibitor of AKT, is under investigation for expanding its therapeutic applicability [14,15]. It inhibits the proliferation of breast cancer cells and breast cancer xenograft models carrying PI3KCA mutations and lacking phosphatase and tension homolog (PTEN) [16]. However, this effect was not observed in clinical trials, which revealed no apparent benefit of AZD5363 in overall or PI3KCA-mutated breast cancer patients [17]. In bladder cancer cells, two AKT inhibitors (MK-2206 and AZ7328) induced apoptosis and reduced the viability of PI3KCA-mutated cells [18,19]. We used a 3D HTS platform to screen drugs and selected AZD5363 as the potential AKT inhibitor in bladder cancer cells carrying PI3KCA mutation.

Everolimus and temsirolimus have been used as mTOR inhibitors and are approved for the treatment of breast cancer, renal cell carcinoma, and pancreatic neuroendocrine tumors [20,21,22]. AZD2014 (vistusertib) is a highly selective mTOR inhibitor [23,24], while BEZ235 (dactolisib) is a dual PI3K/mTOR inhibitor that selectively blocks the signaling of class I PI3K, mTORC1, and mTORC2 [25]. Clinical studies and trials are ongoing to test the efficacy of these drugs in solid and hematologic malignancies. The clinical effectiveness of rapamycin analogues was disappointing in patients with advanced bladder cancer [26]. However, a subset of long-term responders with the mTOR mutation has been identified [27]. In the present study, we selected two bladder cancer cell lines to compare the efficacy of mTOR inhibitors depending on the mTOR mutation status.

Better understanding of the mechanism has not necessarily been translated into improved efficacy of targeted therapy. A potential explanation for the poor response to mTOR inhibitors is the bypass pathway and negative feedback [9]. Therefore, the strategy of the combination therapy that inhibits multiple points in the pathway is critical to improve the response following PI3K/AKT/mTOR pathway blockade. We selected the possible effective combination of inhibitors using a 3D HTS platform and blocked the downstream (mTOR inhibitor) and upstream (AKT inhibitor) mediators of the PI3K/AKT/mTOR pathway in bladder cancer cells. The better efficacy of mTOR inhibitor and AKT inhibitor than other targeted agents was evident, as up to 40% bladder cancer cases showed constitutive activation of the PI3K/AKT/mTOR pathway resulting from either the deletions or mutations of tumor suppressor genes such as PTEN and TSC1 or the amplification or mutations of proto-oncogenes such as PI3KCA and AKT1 [9]. As expected, this study revealed the synergistic response of the combination therapy. Further, this result was observed in specific bladder cancer cells with PI3KCA and mTOR mutations. This observation provides the evidence for the need of trials with the combination of AKT and mTOR inhibitors in patients with PI3KCA- and mTOR-mutated bladder cancers.

The fibroblast growth factor receptors (FGFRs) are receptor tyrosine kinases, signaling various downstream pathways, namely RAS/mitogen-activated protein kinase (MAPK) and PI3K/AKT pathways that mediate cell division and survival [28,29]. Recent studies have demonstrated that FGFR3 alterations commonly occur in up to 21% of locally advanced or metastatic bladder cancer, offering the possibility of new FGFR-targeted therapies [30]. Erdafitinib was the only pan-FGFR inhibitor approved by the FDA (2019) for bladder cancer patients with FGFR2/3 mutations. The overall response rate (ORR) of second-line therapy amounted to 40% (a complete response of 3% and a partial response of 37%) [31]. Pal et al. [32] evaluated the efficacy of BGJ398, an FGFR1–3 inhibitor, in advanced urothelial carcinoma with FGFR3 alterations and reported an ORR of 25.4% and a disease control rate of 64.2%. AZD4547, a FGFR1–3 inhibitor, did not show significant efficacy in gastric cancer [33] and squamous cell lung cancer [34] patients with FGFR alteration. Consistent with previous studies, in the present study, FGFR3 mutation was found in 2 cell lines (29%) among seven bladder cancer cell lines. Of note, using the 3D culture-based HTS platform, we also evaluated the effects of FGFR targeted agents. However, AZD4547 and BGJ398 did not show the efficacy in cell lines with FGFR3 alterations (J82 and SW780 with FGFR3 mutation and RT4 with FGFR3 amplification). Further research is needed to investigate the combination therapy with FGFR inhibitors and other agents to increase efficacy.

We are moving toward precision medicine based on individual genomics by aiming for a more effective, less toxic, and patient-specific treatment. This approach is facilitated by recent advances in next-generation sequencing that allow quick and inexpensive molecular characterization of individual cancers as well as through the development of various targeted agents blocking pathways related to tumor growth [35]. The link between genetic profile and drugs is important as a guide for the treatment of patients with cancer. However, most tumors do not have strong genomic drivers, and responses of drugs may be limited by interactions against multiple regulatory processes. The 3D HTS platform represents a promising, high-throughput, and microscale alternative to conventional in vitro multi-well plate platforms and creates new opportunities for the rapid and inexpensive assessment of drug efficacy at early phases of clinical study for individual cancer patients. Further, our platform offers the advantage of allowing testing of multiple drug combinations.

In conclusion, we demonstrate the synergistic effect of the combination therapy with AKT (AZD5363) and mTOR (AZD2014 and BEZ235) inhibitors against bladder cancer cells with PI3KCA and mTOR mutations. The 3D culture-based HTS platform could serve as a useful preclinical tool to evaluate various drug combinations and may provide valuable insights into precision medicine.

## 4. Materials and Methods

### 4.1. Cell Lines and Cell Culture

Seven bladder cancer cell lines (5637, 235J-BV, J82, SW780, RT4, T24, and UMUC3) were used in this study. The 5637, J82, RT4, T24, and 253J-BV cell lines were purchased from the Korea Cell Line Bank (KCLB, Seoul, Korea), while SW780 and UMUC3 lines were obtained from the American Type Culture Collection (ATCC, Manassas, VA, USA). The 5637, J82, and T24 cells were cultured in Roswell Park Memorial Institute (RPMI)-1640 medium (Gibco, Paisley, UK), and 253J-BV cells were maintained in minimal essential medium (MEM; Gibco). The RT4 cell line was maintained in Dulbecco’s modified Eagle’s medium (DMEM; Gibco), and the SW780 cell line was maintained in L-15 medium (Gibco). All media were supplemented with 10% fetal bovine serum (FBS; Gibco) and a 1% antibiotic anti-mycotic solution (Gibco). All cell lines were incubated at 37 °C in a humidified 5% CO_2_ incubator. Cells were passaged using TrypLE Express (Gibco) for detachment after reaching 80–90% confluency.

### 4.2. HTS of Bladder Cancer Cell Lines on a Micropillar/Microwell Chip Platform

All cell lines were seeded into 3D culture media comprising DMEM F/12 containing 1% antibiotic–antimycotic solution (Gibco), 10 mM HEPES, B27, N2, GlutaMAX (Gibco), 10 nM human gastrin I (Gibco), 1 mM *N*-acetyl-l-cysteine (Sigma Aldrich, St Louis, MO, USA), 10 μg/mL insulin, 20 ng/mL basic fibroblast growth factor (bFGF; Sigma Aldrich), and 50 ng/mL epidermal growth factor (EGF; PeproTech, Rocky Hill, NJ, USA). After 3–5 days, the tissues were dissociated into single cells using accutase (Gibco) and loaded on the micropillar chip.

The layout was designed for screening 12 compounds in a single micropillar/microwell chip, as previously described [36]. In the micropillar chip, approximately 100 cells were immobilized with 0.5% alginate (Sigma Aldrich) in each micropillar. We tested 24 compounds (olaparib, AZD4547, AZD5363, volitinib, selumetinib, AZD1775, everolimus, crizotinib, dasatinib, regorafenib, LJM-716, vemurafenib, cetuximab, vismodegib, dacomitinib, lapatinib, BEZ235, AZD2014, LEE011, staurosporine, neratinib, BGJ-398, and two blind drugs (Selleck Chemicals, Houston, TX, USA)) with six replicates per sample against seven bladder cancer cell lines. A 50 nL droplet of cell/alginate mixture and 950 nL droplet of medium or compounds were dispensed with an S+ microarrayer (S+ Chip Scanner, Samsung Electro-Mechanics Co., Ltd., South Korea). After incubation for 1 day, the micropillar chip containing cells was transferred to a new microwell chip filled with various test compounds, and the combined chips were incubated for 7 days to test the efficacy of each compound, as previously described.

### 4.3. Cell Growth Assessment

For the analysis of viability, the cells were seeded at 3000 cells/well in a 96-well plate, allowed to adhere overnight, and then treated with indicated drugs for 5 days. Cell proliferation inhibition was determined using a CellTiter Glo (Promega, Madison, WI, USA) assay according to the manufacturer’s protocol. The detected luminescence signal was used to calculate the percentage of surviving cells as compared with the control group and to obtain IC50 values.

### 4.4. Colony Formation Assay

Cells were incubated with AZD5363 and an mTOR inhibitor for 2 days. After treatment, cells were harvested, seeded in six-well plates (300 cells/well), and incubated for 10 days. Colonies were assessed using 0.05% crystal violet staining. Colonies with more than 50 cells were counted.

### 4.5. Annexin V assay

Cells (5 × 10^5^ cells) were seeded in a 60-mm dish and incubated for 24 h before treatment with AZD5363 and an mTOR inhibitor for 2 days. After washing twice with phosphate-buffered saline (PBS), the cells were stained using the Annexin V-fluorescein isothiocyanate (FITC)/propidium iodide apoptosis kit (BD Bioscience, San Jose, CA, USA) according to the manufacturer’s instructions. Stained cells were detected and analyzed using FACS verse (BD Bioscience).

### 4.6. Modified Boyden Chamber Cell Invasion Assay

Cells (5 × 10^5^ cells) were seeded in a 60-mm dish and incubated with AZD5363 and an mTOR inhibitor for 2 days. After treatment, cells (1 × 10^5^) were harvested, suspended in a serum-free medium, and loaded onto the top chamber of matrigel-precoated transwell plates (8 μm pore size; Corning Costar, NY, USA). FBS (10%) was used as a chemoattractant in the bottom chamber. After incubation for 24 h, the cells in the bottom chamber were fixed and stained with 0.05% (*w*/*v*) crystal violet. The number of invading cells was quantified in five random fields of each membrane.

### 4.7. Western Blot Analysis

Total cell extracts were obtained using a lysis buffer (20 mM HEPES (pH 7.4), 1% Triton X-100, 1 mM ethylenediaminetetraacetic acid (EDTA), 1 mM magnesium chloride (MgCl_2_), 150 mM sodium chloride (NaCl), 10% glycerol, protease inhibitor, and phosphatase inhibitor cocktail (Invitrogen, Waltham, MA, USA)), and protein concentration was determined using the micro-BCA protein reagent (Pierce Biotechnology, Rockford, IL, USA). Equal amounts of proteins (30 μg) from the clarified lysates were separated by sodium dodecyl sulfate polyacrylamide gel electrophoresis (SDS–PAGE) and transferred onto nitrocellulose membranes (Whatman, Maidstone, UK). The membranes were incubated with antibodies against phospho-AKT (Ser473) (#4060, 1:1000; Cell Signaling Technology (CST), Danvers, MA, USA), AKT (#9272, 1:1000; CST), phospho-mTOR (Ser2448) (#2971, 1:1000; CST), phospho-mTOR (Ser2481) (#2974, 1:1000; CST), mTOR (#2972, 1:1000; CST), phospho-S6 ribosomal protein (Ser2235/236) (#2211, 1:1000; CST), S6 ribosomal protein (#2217, 1:1000; CST), cleaved PARP (#9541, 1:1000; CST), cleaved caspase-3 (#9661, 1:1000; CST), Bcl-2 (#15071, 1:1000; CST), Bax (#5023, 1:1000; CST), phospho-STAT3 (Tyr705) (#9138, 1:1000; CST), STAT3 (#9139, 1:1000; CST), vimentin (#5741, 1:1000; CST), snail (#3879, 1:1000; CST), E-cadherin (#3195, 1:1000; CST), and β-actin (AC-15, 1:5000; Sigma). The enhanced chemiluminescence (ECL) system was used for protein detection (Invitrogen).

## Figures and Tables

**Table 1:** Molecular characteristics of seven bladder cancer cell lines.

Tissue Type	Cell Line	Mutation	Amplification	Deletion	Fusion
Urinary bladder	5637	*TP53*	ERBB3	N/A	N/A
		*RB1*			
		*ERBB2*			
Urinary bladder	J82	*TP53*	N/A	PTEN	N/A
		*PIK3CA*			
		*FGFR3*			
		*RB1*			
		*MTOR*			
		*RET*			
Urinary bladder	SW-780	*FGFR3*	N/A	CDKN2A	N/A
				CDKN2B	
Urinary bladder	RT4	*RhoA*	FGFR3	HRAS	N/A
		*TSC1*	AKT2	CDKN2A	
				CDKN2B	
				MTOR	
Urinary bladder	T24	*TP53*	N/A	N/A	N/A
		*HRAS*			
Urinary bladder	UMUC-3	*KRAS*	N/A	CDKN2A	N/A
		*ERBB3*		CDKN2B	
				PTEN	
				VEGFR	
Urinary bladder	235J-BV	*PIK3CA*	N/A	N/A	N/A
		*ERBB4*			

## Supplementary Materials

Supplementary Materials can be found at https://www.mdpi.com/1422-0067/21/8/2825/s1.

## Abbreviations

3D HTS	three-dimensional high-throughput screening
AKT	protein kinase B
mTOR	mammalian target of rapamycin
PI3K	phosphatidylinositol-3-kinase
Bcl-2	B cell lymphoma-2
PARP	poly (ADP-ribose) polymerase
EMT	epithelial-to-mesenchymal transition
MIBC	muscle-invasive bladder cancer
