# Supplementary material for: Synergistic Effects of Combination Therapy with AKT and mTOR Inhibitors on Bladder Cancer Cells
Source: Int J Mol Sci. 2020 Apr 18;21(8):2825. doi: 10.3390/ijms21082825 (PMC7215775; doi:10.3390/ijms21082825)
Supplement: Supplementary file 1 [file ijms-21-02825-s001.pdf]

# Supplementary Material

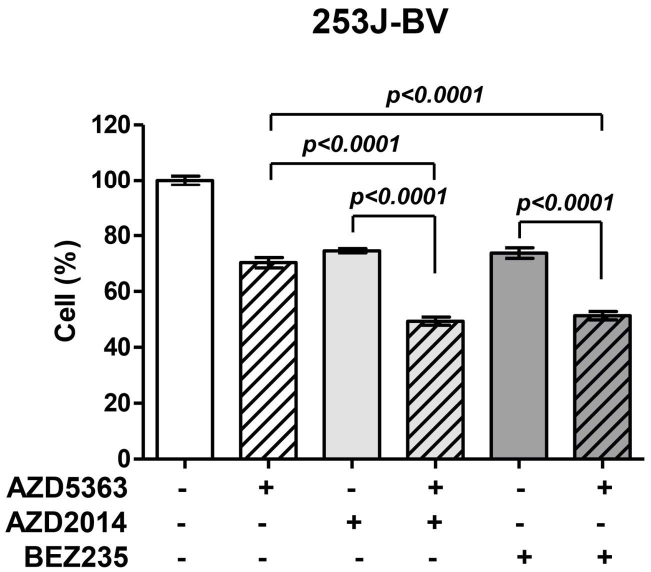


**Figure S1.** Effects of the combination therapy with an AKT inhibitor (AZD5363) and mTOR inhibitors (AZD2014 and BEZ235) on PI3KCA-mutated and mTOR wild-type bladder cancer cells. The effects of the combination of AZD5363 and mTOR inhibitors on bladder cancer cell lines were determined using CellTiter Glo. 253J-BV cells were treated with AZD5363 (3 μM), mTOR inhibitors (1 μM AZD2014 and 0.5 μM BEZ235) or the combination of both for 5 days. The results are presented as the mean ± SD for triplicate wells and are representative of three independent experiments.

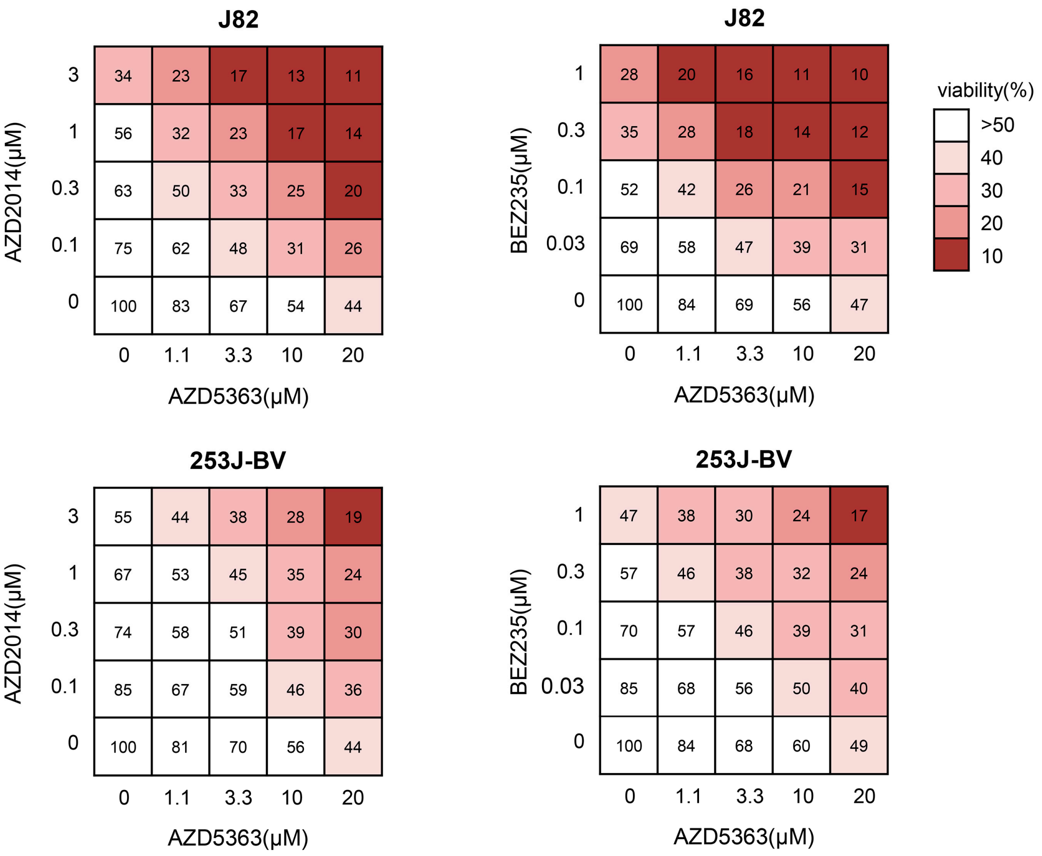


**Figure S2.** Dose-response matrix for a two-drug combination. Matrix block experiment to determine viability, as estimated with the CellTiter Glo assay.
